# Impact of Preeclampsia Severity on Fetal MAPSE and TAPSE: A Prospective Case–Control Study

**DOI:** 10.3390/jcm15082952

**Published:** 2026-04-13

**Authors:** Koray Gök, Merve Baştan, Rahime Tüten, Mustafa Doğan Özçil, Işın Erdoğan, Selçuk Özden, Abdullah Tüten

**Affiliations:** 1 Department of Obstetrics and Gynecology, Cerrahpasa Faculty of Medicine, Istanbul University-Cerrahpasa, 34098 Istanbul, Turkey; drabdtuten@gmail.com; 2 Department of Obstetrics and Gynecology, Sakarya Research and Training Hospital, 54100 Sakarya, Turkey; ymkmerve@gmail.com (M.B.); sozden@sakarya.edu.tr (S.Ö.); 3 Department of Pediatric Cardiology, Bursa City Hospital, 16110 Bursa, Turkey; tutendikmenr@gmail.com; 4 Department of Obstetrics and Gynecology, Faculty of Medicine, Hatay Mustafa Kemal University, 31060 Antakya, Turkey; mustafadoganozcil@gmail.com; 5 Department of Obstetrics and Gynecology, Sanliurfa Education and Training Hospital, 63000 Sanliurfa, Turkey; isin.erdogan78@gmail.com

**Keywords:** preeclampsia, fetus, fetal cardiac function, annular plane systolic excursion, ultrasonography

## Abstract

**Objectives:** The objective of this study was to compare fetal MAPSE and TAPSE values in preeclamptic pregnancies with those in healthy pregnancies and to examine the changes in these parameters according to the severity of preeclampsia. **Methods:** This prospective case–control study enrolled 77 women with preeclampsia and 81 healthy pregnant controls. Fetal MAPSE and TAPSE values were obtained under standardized conditions by experienced operators using M-mode ultrasonography. **Results:** Fetal mitral annular plane systolic excursion (MAPSE) and tricuspid annular plane systolic excursion (TAPSE) values were found to be significantly lower in the preeclampsia group compared with the control group **(*p* < 0.001).** In analyses evaluating preeclampsia cases within themselves, fetal MAPSE and TAPSE values were found to be more significantly reduced in the preeclampsia with severe features group compared to the preeclampsia without severe features group. **Conclusions:** Fetal MAPSE and TAPSE values, measured by M-mode ultrasonography, were found to be significantly lower in the preeclampsia group compared to the control group. The more pronounced decrease in these values, particularly in preeclampsia with severe features cases, suggests that MAPSE and TAPSE measurements may be early indicators of fetal cardiovascular adaptation to the impaired intrauterine environment.

## 1. Introduction

Preeclampsia is a pregnancy-specific disease characterized by new-onset hypertension and proteinuria after the 20th week of gestation [1,2,3]. It complicates 2–8% of pregnancies and is one of the leading causes of maternal and fetal/neonatal morbidity and mortality [2]. Although its pathogenesis is unknown and multifactorial, abnormal placentation and impaired uteroplacental perfusion are regarded as central features of the disease process. The inappropriate intrauterine environment caused by this abnormal placentation activates a number of adaptive mechanisms in both the mother and the fetus. Inadequate trophoblastic invasion and insufficient remodeling of the spiral arteries lead to increased placental vascular resistance, resulting in placental hypoperfusion, oxidative stress, endothelial activation, and an anti-angiogenic/proinflammatory intrauterine environment. Chronic placental dysfunction may alter fetal preload and afterload, reduce oxygen and nutrient delivery, and promote redistribution of the fetal circulation in an attempt to preserve perfusion of vital organs [4,5,6,7].

The heart is the first functional organ to develop during embryogenesis and plays a central role in the fetus’s adaptation to pathological intrauterine conditions [8,9,10]. In order to maintain its function, the heart attempts to adapt in the early stage of any damage by undergoing changes in shape, structure, and size defined as cardiac remodeling [9,10]. Longitudinal myocardial fibers are thought to be especially sensitive to impaired oxygenation, myocardial stress, and changes in loading conditions. Therefore, alterations in longitudinal ventricular motion may become apparent before more global cardiac abnormalities are evident [11,12,13]. For this reason, fetal cardiac assessment is important in various pathological conditions, including preeclampsia.

Many different methods have been described for fetal cardiac functional assessment. Conventional M-mode ultrasonography enables measurement of mitral annular plane systolic excursion (MAPSE) and tricuspid annular plane systolic excursion (TAPSE), parameters that reflect longitudinal ventricular systolic excursion and may provide indirect information about fetal myocardial mechanics. These parameters offer several practical advantages: they are relatively easy to obtain, can be measured quickly, are widely available, and can be incorporated into routine fetal cardiac assessment without advanced software or complex post-processing. However, MAPSE and TAPSE should be interpreted as indirect measures of longitudinal ventricular motion rather than as definitive evidence of global fetal cardiac dysfunction [14,15,16]. Although there are studies examining the use of these parameters in various disease groups during the antenatal period [17,18,19], there are few studies that evaluate these parameters together in pregnancies complicated by preeclampsia, and the existing findings are not entirely consistent. Furthermore, the relationship between preeclampsia severity and these M-mode parameters does not appear to be sufficiently clear [12,20,21].

Therefore, this study was planned to address a focused pathophysiological question rather than a diagnostic or prognostic one. Our study aimed to compare fetal MAPSE and TAPSE values in preeclamptic pregnancies with those in healthy pregnancies and to examine the changes in these parameters according to the severity of preeclampsia.

## 2. Materials and Methods

This prospective case–control study included 77 pregnant women diagnosed with preeclampsia who were admitted to the Perinatology Department of the Obstetrics and Gynecology Clinic, Sakarya University Training and Research Hospital, between February 2021 and February 2022 and delivered at the same institution. The control group consisted of 81 healthy pregnant women with comparable gestational age and no obstetric complications. Clinical and obstetric data were obtained from hospital records. The study protocol was approved by the Local Ethics Committee of Sakarya University Faculty of Medicine (approval no. E16214662-050.01.04-6813-15; 5 February 2021).

Pregnancies were excluded if any of the following were present: multiple gestation, gestational or pregestational diabetes mellitus, chronic maternal systemic disease including chronic hypertension, chronic liver disease, chronic kidney disease, or rheumatologic disorders, as well as acute or chronic infection. Additional exclusion criteria were preterm premature rupture of membranes, chorioamnionitis, fetal structural or chromosomal abnormalities, Doppler findings suggestive of placental insufficiency, sonographic estimated fetal weight below the 10th percentile, and maternal use of acetylsalicylic acid, corticosteroids, or heparin.

Preeclampsia was defined as new-onset hypertension after 20 weeks of gestation in the presence of proteinuria. Hypertension was defined as systolic blood pressure ≥ 140 mmHg and/or diastolic blood pressure ≥ 90 mmHg. Proteinuria was defined as ≥300 mg in a 24 h urine collection, a protein-to-creatinine ratio ≥ 0.3, or a dipstick reading of 2+ or greater. Patients were classified as having **preeclampsia without severe features** if they met the diagnostic criteria for preeclampsia in the absence of severe clinical or laboratory findings. **Preeclampsia with severe features** was diagnosed in women with systolic blood pressure ≥ 160 mmHg and/or diastolic blood pressure ≥ 110 mmHg. In the absence of severe-range blood pressure, patients were also classified as having preeclampsia with severe features if one or more of the following were present: new-onset headache, visual disturbances, or other central nervous system symptoms; persistent right upper quadrant or epigastric pain; elevated liver enzyme levels, particularly alanine aminotransferase or aspartate aminotransferase; renal insufficiency, defined as urinary protein excretion ≥ 2 g/24 h or serum creatinine > 10^6^ μmol/L; thrombocytopenia, defined as a platelet count < 100 × 10^9^/L; or pulmonary edema [1].

All ultrasonographic examinations were performed by two experienced sonographers (KG and MB) using Voluson 730 and Voluson E6 ultrasound systems (GE Medical Systems, Milwaukee, WI, USA). Sonographic assessment was completed before initiation of antihypertensive treatment and/or magnesium sulfate administration. All measurements were obtained during periods without fetal movement. Fetal mitral annular plane systolic excursion (MAPSE) and tricuspid annular plane systolic excursion (TAPSE) were measured according to previously published and validated protocols [19].

Statistical analyses were performed using SPSS version 24.0 (SPSS Inc. and Lead Tech Inc., Chicago, IL, USA). Normality of data distribution was assessed using the Kolmogorov–Smirnov test. Normally distributed variables were expressed as the mean ± standard deviation, whereas non-normally distributed variables were presented as the median and interquartile range. Comparisons between two independent groups were performed using the independent-samples *t* test for parametric variables and the Mann–Whitney U test for non-parametric variables. Comparisons among multiple groups were conducted using the Kruskal–Wallis test followed by Bonferroni-corrected post hoc analysis when appropriate. A *p* value < 0.05 was considered statistically significant.

## 3. Results

The comparison of the characteristics of preeclamptic cases and the control group is presented in Table 1. No statistically significant difference was found between the preeclamptic cases and the control group in terms of age, body mass index (BMI), and gestational week at which the measurements were performed. Fetal MAPSE and TAPSE values were found to be statistically significantly lower in preeclamptic cases compared to the control group (*p* < 0.001).

Fetal MAPSE and TAPSE values were evaluated among the groups of preeclampsia without severe features, preeclampsia with severe features, and the control group using the Kruskal–Wallis test, and a statistically significant difference was found between the groups (*p* < 0.001) (Table 2). Subsequent pairwise comparisons using the Mann–Whitney U test demonstrated that fetal MAPSE and TAPSE values differed significantly between all three groups (*p* < 0.001).

## 4. Discussion

In this prospective case–control study, we found that fetal MAPSE and TAPSE values were lower in preeclamptic pregnancies than in the control group. Furthermore, when the preeclamptic group was evaluated within itself, these values were found to be lower in the subgroup with preeclampsia with severe features. These findings suggest that longitudinal ventricular motion is altered in fetuses exposed to the adverse intrauterine environment of preeclampsia.

Why did we focus on fetal MAPSE and TAPSE measurements in fetal cardiac assessment in this study? More advanced techniques, such as tissue Doppler imaging, speckle-track echocardiography, strain analysis, and velocity vector imaging, can detect earlier and more subtle myocardial changes and provide better differentiation between altered adaptation and true functional impairment. However, these methods are not always available in routine clinical settings and may be more technically demanding [21,22]. Longitudinal cardiac motion is determined by the longitudinal myocardial fibers, which are the most sensitive to conditions such as hypoxia and pressure/volume overload, and it is considered one of the earliest and most sensitive indicators of cardiac dysfunction [11,12,13]. Maximum mitral and tricuspid annular plane systolic excursions (MAPSE and TAPSE, respectively) can be measured by M-mode ultrasonography, which is a simple, sensitive, and reproducible method, and these parameters may provide information about the longitudinal motion of the fetal heart [23]. For this reason, we preferred fetal MAPSE and TAPSE parameters to evaluate fetal cardiac assessment in preeclampsia, which is accompanied by an intrauterine hypoxic environment.

In the literature, fetal MAPSE and TAPSE values have been studied in various complicated pregnancies, and it has been shown that these values may be sensitive to adverse intrauterine environments. In two separate studies conducted in monochorionic twin pregnancies complicated by twin-to-twin transfusion syndrome, decreased MAPSE and TAPSE values were reported in both recipient and donor fetuses, suggesting cardiac dysfunction [24,25]. Guo et al. found lower fetal MAPSE and TAPSE Z-scores in fetuses with heart failure compared with normal fetuses and stated that these scores may be used as markers both for assessing systolic cardiac function and for determining the severity of heart failure in affected fetuses [18]. In studies involving diabetic pregnancies, however, the results have been heterogeneous; while some studies reported no significant difference in fetal MAPSE and TAPSE values [26,27], another study, in contrast, reported decreased values in diabetic pregnancies [17]. In a study evaluating fetal cardiac function by M-mode ultrasonography in fetuses with intrauterine growth restriction (IUGR/FGR), fetal MAPSE and TAPSE values were reported to be significantly lower than those in the control group, and the investigators suggested that these parameters may be sensitive indicators for detecting cardiac dysfunction related to placental insufficiency [19]. Both fetal growth restriction and preeclampsia share similar pathophysiological mechanisms in which placental insufficiency plays a central role. In addition, there are studies demonstrating fetal cardiovascular remodeling and dysfunction in pregnancies complicated by isolated fetal growth restriction [28,29]. Therefore, because it could affect the study outcomes, we did not include preeclamptic cases accompanied by fetal growth restriction (FGR).

The first study to evaluate fetal MAPSE and TAPSE together in preeclamptic pregnancies was conducted by Balli et al. In that study, unlike ours, only patients with mild preeclampsia were included, and, again unlike our study, the pregnancies were evaluated at 33 and 34 weeks of gestation in the study and control groups, respectively [20]. In that study, which compared 65 women with mild preeclampsia and 55 healthy pregnant women, no significant difference was found between the groups. The authors reported that no change in ventricular systolic function could be demonstrated by M-mode ultrasonography but that tissue Doppler ultrasonography might reveal early subclinical changes in systolic and diastolic fetal cardiac function due to the increased afterload observed in preeclampsia. Subsequently, in another study by Yu et al. [21], 104 pregnant women, including 34 with gestational hypertension, 32 with preeclampsia, and 38 controls, were evaluated at a mean gestational age of 31 weeks. In that study, in which no evaluation was made according to the severity of preeclampsia, fetal MAPSE and TAPSE values measured by M-mode ultrasonography were lower in the preeclampsia group; however, no significant difference was found between the groups, and ventricular functions were reported to be similar. Nevertheless, evaluation by velocity vector imaging echocardiography demonstrated impaired fetal ventricular function, and the authors suggested that this method may be superior to conventional echocardiography for assessing fetal cardiac function in preeclampsia [21]. More recently, in a study by Hendem et al. investigating fetal cardiac functions in preeclamptic pregnancies, 48 healthy pregnant women and 48 women with preeclampsia were compared at 32–34 weeks of gestation; fetal MAPSE and TAPSE values were reported to be lower in the preeclampsia group than in the control group [12]. In that study, similar to ours, an evaluation according to the severity of preeclampsia was performed, but unlike our study, fewer patients were included (19 with mild and 29 with severe preeclampsia), and fetal MAPSE and TAPSE values did not differ between the mild and severe groups. In the same study, it was emphasized that tissue Doppler imaging could demonstrate subclinical functional changes in the fetal heart earlier and more sensitively in preeclamptic pregnancies; this finding is consistent with the results of Balli et al. [12,20]. Among these studies, only the study by Yu et al. [21], similar to ours, reported comparable BMI values between the study and control groups, whereas BMI was not specified in the other two studies [12,20]. In our study, the groups were composed of subjects with similar BMI values. Moreover, similar to the studies by Yu et al. [21] and Hendem et al. [12], but unlike that of Balli et al. [20], we found reduced fetal MAPSE and TAPSE values. In addition, contrary to the study by Hendem et al. [12], we found that fetal MAPSE and TAPSE values decreased even more significantly as the severity of preeclampsia increased. In our study, patients with preeclampsia were divided into subgroups with and without severe features at a specific gestational age, and unlike previous studies, a larger number of patients were included. This allowed our findings to be obtained in a more homogeneous population. These methodological differences may explain the discrepancy between our findings and some of the conflicting results in the literature.

What may be the possible explanation for the fetal cardiovascular response manifested by decreased fetal MAPSE and TAPSE values in preeclamptic pregnancies in this study? Although the exact cause of preeclampsia remains unknown, its pathophysiology is highly complex. However, defective uteroplacental function, which manifests early in pregnancy as impaired placental vascular transformation and consequent increased placental resistance, lies at the center of the pathogenesis of early-onset preeclampsia [30]. The placenta is the central organ for nutrient and gas exchange throughout pregnancy and plays a critical role in fetal growth and development. Preeclampsia is characterized by abnormal placentation, and cardiotoxic products such as antiangiogenic factors and reactive oxygen species generated by oxidative stress circulate in the blood of preeclamptic women [28]. It has been proposed that this unfavorable intrauterine hypoxic environment in preeclampsia limits the availability of substrates necessary for fetal growth and may also lead to structural and functional impairment of the fetal heart [12,28,30]. The fetal heart is the primary organ that mediates adaptation to the deteriorating intrauterine environment [9], and in this process, the first structures to be affected are the longitudinal myocardial fibers [11]. Therefore, we believe that decreased fetal MAPSE and TAPSE values, which provide information about longitudinal cardiac motion, are an expected finding in preeclamptic cases. In light of all these findings, the fetal cardiovascular response in preeclamptic pregnancies, particularly the lower MAPSE and TAPSE values observed in the group with severe features as in our study, can be explained by the underlying pathophysiology of the disease.

The present study has several strengths, including its prospective design, the comparable demographic characteristics of the study groups, and the stratification of preeclampsia according to disease severity. However, several limitations should be considered when interpreting the findings. First, the observational case–control design does not permit causal inference. Second, because neonatal outcomes, perinatal complications, and postnatal echocardiographic follow-up data were unavailable, it cannot be determined whether lower MAPSE and TAPSE values reflect adaptive physiology, subclinical myocardial involvement, or nonspecific hemodynamic variation. Third, the absence of intraobserver and interobserver reproducibility analyses constitutes an important methodological limitation, particularly for operator-dependent M-mode measurements. Fourth, the evaluation was restricted to M-mode parameters, whereas more advanced techniques such as tissue Doppler imaging, speckle-tracking echocardiography, and strain-based methods were not included. Fifth, no multivariable analysis and no prespecified sample size calculation were available. Sixth, although subgroup analysis according to disease severity is a strength of the study, the relatively limited number of patients in the severe-features subgroup may reduce the robustness of subgroup-based conclusions. Finally, although the differences between groups were statistically significant, statistical significance alone should not be interpreted as evidence of clinical utility. The absolute magnitude of the observed differences, the possible overlap between group distributions, and the absence of outcome-based validation limit the immediate clinical interpretability of MAPSE and TAPSE in this setting.

## 5. Conclusions

Fetal MAPSE and TAPSE values were significantly lower in preeclamptic pregnancies than in healthy pregnancies in this study. Moreover, this reduction appeared to be more pronounced in the preeclampsia group with severe features. These findings suggest that longitudinal ventricular motion is altered in fetuses exposed to the adverse intrauterine environment of preeclampsia. However, because MAPSE and TAPSE are surrogate markers and were not linked to postnatal functional validation or clinically relevant outcomes in this study, they should be interpreted cautiously. Prospective studies with larger sample sizes, multicenter designs, and postnatal cardiac follow-up are needed to more clearly define the prognostic value and clinical role of these parameters in decision-making processes.

## Figures and Tables

**Table 1:** **Comparison of characteristics, fetal MAPSE and TAPSE between preeclamptic cases and control group.**

Variables	Preeclampsia Group (n: 77)	Control Group (n: 81)	*p*-Value
**Maternal age (years)**	31 (18–43)	29 (19–42)	0.289
**Body mass index (BMI) (kg/m^2^)**	26.48 ± 1.29	26.19 ± 1.59	0.194
**Gestational age at the time of the study (weeks)**	30.56 (29.14–32)	30.42 (29.28–32)	0.640
**MAPSE (mm)**	4 (3.6–5.1)	5.5 (4.7–5.8)	**<0.001**
**TAPSE (mm)**	5.7 (5.2–6.2)	6.8 (6–7.5)	**<0.001**

Data are expressed as median (minimum-maximum). *The bold value p*  <  0.05 indicates a significant difference.

**Table 2:** **Kruskal–Wallis test comparing fetal MAPSE and TAPSE between Preeclampsia without severe features and Preeclampsia with severe features and control group.**

Variables	Control Group (n: 81)	Preeclampsia Without Severe Features (n: 46)	Preeclampsia with Severe Features (n: 31)	*p*-Value
**MAPSE (mm)**	5.5 (4.7–5.8)	4.4 (3.8–5.1)	3.8 (3.6–4)	**<0.001**
**TAPSE (mm)**	6.8 (6–7.5)	5.85 (5.6–6.2)	5.4 (5.2–5.7)	**<0.001**

Data are expressed as median (minimum-maximum). *The bold value p*  <  0.05 indicates a significant difference.

## Data Availability

The datasets generated and/or analyzed during the current study are used in the paper.
